# How the risk of liver cancer changes after alcohol cessation: A review and meta-analysis of the current literature

**DOI:** 10.1186/1471-2407-11-446

**Published:** 2011-10-13

**Authors:** Gawain A Heckley, Johan Jarl, Benedict O Asamoah, Ulf G-Gerdtham

**Affiliations:** 1 Health Economics & Management, Institute of Economic Research, Lund University, Box 117, 22100, Sweden; 2 Center for Primary Health Care Research, Skåne University Hospital, Malmö, Lund University/Region Skåne, SE-20502 Malmö, Sweden; 3 Economics Department, Lund University, Box 117, 22100, Sweden

## Abstract

**Background:**

It is well established that drinking alcohol raises the risk of liver cancer (hepatocellular carcinoma). However, it has not been sufficiently established as to whether or not drinking cessation subsequently reduces the risk of liver cancer and if it does reduce the risk how long it takes for this heightened risk to fall to that of never drinkers. This question is important for effective policy design and evaluation, to establish causality and for motivational treatments.

**Methods:**

A systematic review and meta-analysis using the current available evidence and a specific form of Generalised Least Squares is performed to assess how the risk of liver cancer changes with time for former drinkers.

**Results:**

Four studies are found to have quantified the effect of drinking cessation on the risk of liver cancer. The meta-analysis suggests that the risk of liver cancer does indeed fall after cessation by 6-7% a year, but there remains a large uncertainty around this estimate both statistically and in its interpretation. As an illustration it is estimated that a time period of 23 years is required after drinking cessation, with a correspondingly large 95% confidence interval of 14 to 70 years, for the risk of liver cancer to be equal to that of never drinkers.

**Conclusion:**

This is a relatively under researched area and this is reflected in the uncertainty of the findings. It is our view that it is not possible to extrapolate the results found here to the general population. Too few studies have addressed this question and of the studies that have, all have significant limitations. The key issue amongst the relevant studies is that it appears that current drinkers, abstainers and former drinkers are not composed of, or effectively adjusted to be, similar populations making inferences about risk changes impossible. This is a very difficult area to study effectively, but it is an important topic. More work is required to reduce both statistical uncertainty and tackle the various study limitations this paper highlights and until this is done, the current result should be considered preliminary.

## Background

Several studies in different countries have investigated the relationship between alcohol cessation and the risk of liver cancer that confirm the association of higher alcohol consumption and increased risk of liver cancer (also known as hepatocellular carcinoma)[1-3]. The Gutjahr *et al*. overview and meta-analysis[4] reports that relative risks of liver cancer for low, hazardous and harmful levels of alcohol consumption compared to never drinkers are 1.45, 3.03, and 3.60 respectively (Where low consumption = (0-19.90 grams (females), 0-39.99 grams (males) pure alcohol per day), hazardous consumption = (females 20-39.99 grams, males 40-59.99 grams), harmful consumption = (females 40+ grams, males 60+ grams)). Whilst it is established that alcohol increases the risk of liver cancer, it is not established to what extent this increased risk is reversible through abstention of alcohol consumption.

In addition to confirming whether or not alcohol has a causal detrimental impact on the risk of liver cancer, it is also interesting from a policy perspective to understand which policies will best reduce the increased risk of liver cancer due to alcohol consumption. Evidence of reversibility will mean effective policies with the aim of reducing consumption will have a positive impact. If alcohol induced risk is not reversible (in a relatively short period of time) it will be better to focus on prevention. Evidence of reversibility could also be important for motivational treatments.

This paper forms part of a larger project investigating the avoidable cost of alcohol. To estimate the avoidable cost of alcohol it is necessary to understand the way in which risk declines (or increases) post alcohol consumption cessation/reduction. There exists evidence on the impact of alcohol cessation on liver cirrhosis. Jarl et al. 2010[5] show that from the point of alcohol cessation there is a lag of around 20 years for men and 23 years for women for the heightened risk of liver cirrhosis to fall to that of never drinkers. It is then calculated that 72% of the total attributable cost of alcohol caused liver cirrhosis could potentially be avoided[5]. Similarly Rehm et al 2007[6] investigate the temporal sequence and the strength of the association between the risk of oesophageal, head and neck cancer changes and alcohol cessation. However, there currently does not exist a summary of the evidence of the impact of alcohol cessation on liver cancer. It is also hoped that highlighting the question of reversibility will spawn further research in this area.

The purpose of this study is to assess to what extent alcohol-related-elevated-risk of liver cancer is reversible and, if so, how many years after drinking cessation it would take for the elevated-risk of cancer to fall back to that of never drinkers. This paper addresses the lack of an overview of the evidence base in this area by performing both a systematic literature review of the current epidemiological research and a meta-analysis of the results from the systematic review. The first section sets out the methodology of the systematic review, the data extraction process and the meta-analysis methodology. The meta-analysis results are then presented. The paper concludes with a discussion of both the systematic review findings and the results from the meta-analysis.

## Methods

### Literature search

A systematic literature search was conducted independently by one author in June and July, 2010 to review and summarise epidemiological studies on effects of alcohol cessation on the risk of liver cancer (ICD 10 code C22). The results were then independently verified by another author and updated to May 2011. Both the PRISMA 2009 checklist and MOOSE statement 2000 checklist for preferred reporting items have been followed. The search terms below were used.

["alcohol" AND ("liver cancer" OR "liver cell carcinoma" OR "malignant neoplasm of liver" OR "hepatocellular carcinoma" OR "malignant hepatoma" OR "liver sarcoma" OR "hepatoblastoma" OR "hepatoma" OR "liver angiosarcoma" OR "intrahepatic bile duct carcinoma" OR "cholangiocarcinoma" OR "kupffer cell sarcoma") AND ("risk" OR "association" OR "relationship" OR "relation" OR "correlation" OR "connection" OR "link") AND ("cessation" OR "quit drinking" OR "quitting drinking" OR "stop drinking" OR "stopping drinking" OR "abstainers" OR "abstinence" OR "ex-drinkers" OR "former drinkers" OR "withdrawal" OR "withdraw" OR "cease drinking" OR "give up drinking" OR "discontinue drinking" OR "halt drinking")]. Liver cancer was again replaced with ICD 10 code C22 in another search. The search was initially performed in PUBMED and MEDLINE.

### Selection of studies

The following exclusion criteria were applied; (1) the study was not published in English; (2) the study was not performed on humans; (3) the study was not designed to capture data on alcohol cessation (ex-drinkers) or did not distinguish between lifetime abstainers and former drinkers (those who had quit drinking and those who never drank alcohol); and (4) the study did not investigate cancer of the liver.

**Figure 1:**
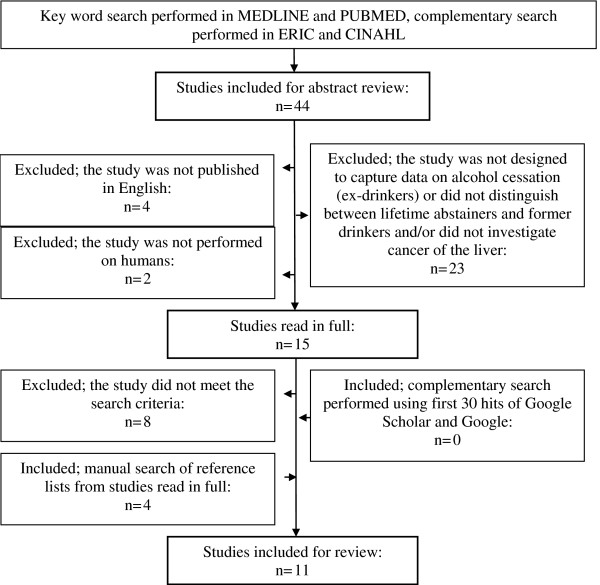
**Flow diagram of the selection process for the papers selected to be included in the systematic review**.

The search initially yielded 44 articles in PUBMED and MEDLINE. Inspection of abstracts narrowed it down to 15 articles potentially relevant to the current study area. These articles were read in full and the reference lists were searched manually. Complementary search was done in ERIC, CINAHL, Google scholar and Google. In all, eleven relevant articles were obtained that met the set criteria (above) to be included in the review and are summarised and compared in Additional file 1. This is shown schematically in a flow diagram in Figure 1. Of the eleven relevant studies that look at the effect of drinking cessation on the risk of liver cancer, five capture data on the duration of cessation.

### Data extraction

Five relevant studies were identified by the systematic review that quantified the risk impact on liver cancer of drinking cessation and the duration of drinking cessation. In two studies (Ozasa and Ogimoto *et al*.)[7,8] the same data was partially used. In order not to give inappropriate weight to this particular dataset, only the most recent study was included in the meta-analysis, leaving four studies. The data is extracted in order to summarise what the available evidence suggests. It should be acknowledged that any findings must be interpreted with caution due to the limited amount of evidence available on this topic.

Information extracted from selected articles includes but is not limited to; country and study years, study type, sample size, age, gender specific calculations, statistical methods, whether incidence of liver cancer was confirmed by histology, definition of a former drinker and risk estimates with confidence intervals (see Additional file 1).

Drinking cessation was presented in categories in the studies. To convert this into a "dose" measure the mid points of the categories were used. Where the category was open ended the same interval width as the previous category was used. In some studies "never drinker" was used as the reference category in the risk estimation. For such studies, the risk estimates were re-calculated using "current drinker" as the reference category on the assumption that just quitters are best matched to current drinkers as a reference group. Standard errors and cell numbers used to calculate the adjusted odds ratios in the original papers were imputed using the method set out in Hamling et al 2008[9]. Confidence intervals (at the 95% level) were then re-calculated for the papers where the reference group was changed to current drinkers.

An additional dataset was also created where those who just quit drinking are used assuming that the observed risk at first period after cessation is equal to the risk at point of cessation. Again, the standard errors and 95% confidence intervals are calculated based on the imputed cell numbers derived from the Hamling et al. 2008[9] approach. This additional dataset was created because although using current drinkers as the reference group has a clear logic and message, it is often the case that the former drinker category has a different composition to that of current drinkers[10]. In order to determine how risk declines after alcohol cessation it is necessary for treatment and control groups to be comparable to be able to make any robust conclusions. If the two groups are different and these differences have not been effectively controlled for then it isn't possible to make any robust conclusions about how the risk of liver cancer changes after alcohol consumption.

### Meta-analysis - Statistical methodology for trend estimation

The effect measure of interest is the relative risk (RR) of liver cancer as this is the most appropriate for the cost of alcohol literature. However, some studies report odds ratios (ORs) and others present hazard ratios (HRs). At low prevalence levels it can be assumed that RR, OR and HR are approximately equal. Due to the nature of liver cancer and its low prevalence amongst the population all measures will be treated as ORs. This allows for greater mathematical ease whilst still allowing the interpretation in terms of relative risks.

The problem of bias amongst published studies, in particular the issue of publication bias, is considered through the utilisation of funnel plots of the data, with current drinkers as the reference group. This is a helpful tool although other interpretations other than publication bias are not excluded. See Egger, M. *et al*.[11] for a comprehensive discussion of potential sources of bias in meta-analysis.

In traditional meta-analysis the risks are weighted by the inverse of their variance (Weighted Least Squares) with the assumption that the variances are independent. However, it cannot be realistically assumed that the variance of the odds ratios by duration are independent when they are all estimated using a common reference group within each study. Ignoring this underestimates the variance of the slope and consequently will lead to spurious accuracy of reported standard errors. A proposed strategy to deal with this is suggested by Greenland *et al*.[12] whereby only the summary estimates and marginal data are required to get an efficient estimate. This utilises a Generalised Least Squares (GLS) approach using an iteratively estimated variance covariance matrix to scale the estimate. The application of the adjusted variance covariance matrix accounts for the higher true variance in the estimates to give a more accurate picture of the statistical uncertainty. This approach is adopted and all dose-response information is pooled prior to estimation.

In estimating the trend of years since cessation of alcohol (dose) and the log risk of liver cancer, additional explanatory variables will be utilised including a squared term to test whether a nonlinear association between log odds ratio of liver cancer and duration of alcohol cessation exists as well as controls for heterogeneity between and within studies. This approach is called the "fixed effects meta-regression" model, which assumes that each study is estimating the same underlying trend. After controlling for study specific differences, the errors are assumed to have a zero mean distributed with normal variance:

$${\text{Y}}_{\text{it}}={\text{X}}_{\text{it}}\beta +{\text{e}}_{\text{it}}\;\text{where}\;{\text{e}}_{\text{it}}\sim \text{N}\left(0,\sigma_{\text{i}}^{2}\right)$$

Where β is a k × 1 vector of coefficients and X_it _is a t × k matrix of k covariates for study i, dose period t. When applying the fixed effects meta-regression model using GLS on dose-response data a test for heterogeneity is the Q test:

$$\text{Q}={\left(\text{Y}-\text{Z}\beta \right)}^{\prime }\Sigma^{-1}\left(\text{Y}-\text{X}\beta \right)$$

where Σ is the estimated variance covariance matrix estimated using the method proposed by Greenland *et al*. in the GLS estimate[12]. This is evaluated using the chi-square distribution, n-k degrees of freedom (k is the number of coefficients) with the null hypothesis that the model is fitted correctly and there is no unexplained between-study heterogeneity. If the null is not rejected, due to low power, it can only be said that the statistic could not detect any significant between-study heterogeneity, not that there is no heterogeneity[13].

## Results

**Figure 2:**
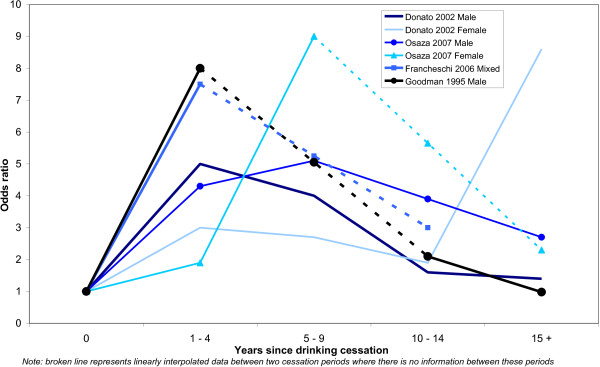
**Odds ratio for former drinkers by duration of cessation, current drinkers as reference category**.

This section focuses on the results of the meta-analysis. The results should be considered preliminary due to the limited available evidence in this area. The results of the systematic literature review are presented in Additional file 1. Figure 2 depicts the dose-response relationship as found in the four studies appropriate for inclusion in the meta-analysis. The combined total number of observations is 19 where the reference group is current drinkers and 13 where the reference group is just quitters. All studies show an increase in risk for recent quitters of alcohol compared to current drinkers. There is however, a clear decline in risk beyond the first measured point after cessation with the exception of the findings for women (it should be noted that both female groups are based on very small samples).

**Figure 3:**
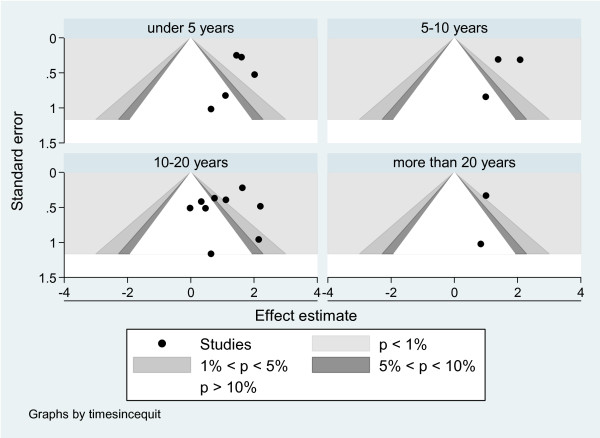
**An assessment for publication bias - the funnel plot test**.

Figure 3 graphically illustrates the association between significance of results and effect size in the included studies. It is not clear that there is any bias as the results are in the wrong direction to what medical science would expect and there are equally as many non-significant results as significant results. There is little to indicate that publication bias is an issue.

Table 1 presents the results from the GLS meta-analysis trend estimation regressions. Models 1-3 are applied to the data with current drinkers as the reference group and estimate a log linear, then a log non linear relationship between time since cessation (dose) and the risk of liver cancer. There is a clear inverse squared relationship between risk of liver cancer and time, capturing the observed higher risk of those who have just quit drinking compared to current drinkers and the subsequent fall in risk over time for the former drinker group. Individually dummies for gender, between-study differences (Study characteristics) and radiation (which controls for one study performed on a population sample from Nagasaki and Hiroshima) all show significance (results not shown). However, the trend coefficients become non-significant when a dummy variable is used to allow for a structural break between the first ten years of cessation and subsequent years. The regression is unable to fit a trend when a structural break is included to account for the higher risk of just quitters, and in many of the studies former drinkers in general compared to current drinkers. This implies that the only statistically significant relationship that we observe is that former drinkers who recently quit drinking have a higher risk of liver cancer than current drinkers in these studies. According to the Q statistic, accounting for the higher risk of former drinkers in the first measured period of cessation accounts for most of the heterogeneity between the studies.

**Table 1:** Meta-analysis results: Change in odds ratio of liver cancer after an additional year not drinking

	*Compared to current drinkers*				Compared to just quitters	
	
Model	*1*	2	3	4	5	6
Dose (A year of not drinking)	**1.08**	**1.40**	1.07	**0.94**	**0.93**	**0.93**
Dose squared		**0.98**	1.00			
**Controls for:**						
Study characteristics*	No	No	No	No	Yes	Yes
Radiation fall out	No	No	No	No	No	**Yes**
Quit in last ten years	No	No	**Yes**	No	No	No
Dose*Study characteristics	No	No	No	No	No	Yes
Q statistic ^#^	144.3	55.8	**15.9**	**19.0**	**18.1**	**6.6**

*Study characteristics = 1 if the study was in Japan, a prospective cohort design, had no minimum gap between giving up drinking and joining study, and no controls for hepatitis. # pr>Chi2 that there is no heterogeneity according to the Q statistic. Bold represents statistically significant figures at the 95% confidence level, or Q statistic finds no heterogeneity

Models 4-6 are applied to the data where former drinkers who have just quit (shortest recorded cessation period) are used as the reference group and all other former drinker categories of longer duration are compared to this group. Structural heterogeneity and/or influences on the trend estimate of risk decline of liver cancer after drinking cessation are explored. Model 4 is a simple regression of dose and log risk and finds a reversible effect of drinking cessation on the risk of liver cancer. It is not clear that there is a non-linear relationship between the log odds ratio of liver cancer and duration of alcohol cessation because including a second polynomial makes the linear dose term non-significant (results not shown). The second polynomial is therefore dropped. Gender no longer has a structural impact on the estimates which is not surprising given the very small samples of the female estimates (again results not shown). Only the dummy "Radiation" is significant. The "study characteristics" dummy is tested to see if it influences the trend. There is no evidence that the trend estimates are different between study types. The results from models 4-6 all show consistent trend estimates and indicate no measureable between study heterogeneity as per the Q statistic suggesting this is a robust relationship and a common trend exists between the studies.

### Sensitivity analysis

#### Testing the importance of how dose is coded

Table 2 shows the results from some sensitivity analyses assessing whether or not the results presented here are sensitive to how dose is coded for the meta-analysis regression. Both models 3 and 6 are re-runs using the lowest value from each dose category rather than the middle value. The results do not differ substantially given quite a dramatic difference in how dose is coded. This suggests that the original estimates found from Models 3 and 6 are quite robust relationships and not sensitive to coding options.

**Table 2:** Sensitivity analysis of meta-analysis to the coding of years since cessation

Model:	*3 (original results)*	*3 (New dose coding)*	*6 (original results)*	*6 (New dose coding)*
Dose (1 year of not drinking)	1.07	1.00	**0.93**	**0.92**
Dose squared	1.00	1.00		
**Controls for:**				
Study characteristics*	No	No	Yes	Yes
Radiation	No	No	**Yes**	**Yes**
Quit in last ten years	**Yes**	**Yes**	No	No
years since cessation*heterogeneity	No	No	Yes	Yes
Q statistic ^#^	**15.9**	**18.38**	**6.6**	**7.95**

* Study characteristics = 1 if the study was in Japan, a prospective cohort design, had no minimum gap between giving up drinking and joining study, and no controls for hepatitis. # pr>Chi2 that there is no heterogeneity according to the Q statistic. Bold represents statistically significant figures at the 95% confidence level, or Q statistic finds no heterogeneity

## Discussion

Across all of the studies two types of study design were used, either case-control or prospective cohort. All of the eleven studies found in the systematic review show that the risk of liver cancer just after alcohol cessation is either the same or higher compared to current drinkers. Given the detrimental effect of alcohol, an a priori expectation was that the risk would be lower after cessation, all else equal.

Due to the nature of case-control and prospective cohort studies, matching of treatment and control groups and/or controlling for confounding factors such as smoking, diabetes and past drinking habits (and other factors known to have an influence on risk of liver cancer) are important to ensure the effect of alcohol cessation is being isolated. Indeed some of the studies presented in the systematic review show that smoking matters and that there is an interaction effect between smoking and alcohol consumption[14], but no study looks to control for both duration of alcohol cessation and smoking habits. The amount of alcohol consumed prior to cessation is expected to influence the risk of liver cancer too[4], yet none of the studies presented here control for this when comparing treatment and control groups. Diabetes is also commonly looked at but rarely at the same time as the effect of cessation of alcohol consumption on the risk of liver cancer.

However, beyond showing that various factors increase the risk, none of the studies presented here control for all of them when considering the time effect of alcohol cessation on liver cancer. It is therefore not clear that these studies are able to isolate the average treatment effect of alcohol cessation on the risk of liver cancer. The design of the studies and the lack of effective controls for confounding factors (possibly as a consequence of lack of focus on the question at hand) are likely to bias the end results. It means that the reader cannot be sure whether there is a real relationship between the observed risk of treatment and control groups or whether this is due to some other unobserved or uncontrolled for factors.

The problem is particularly acute here where the medical assumption of reversibility would predict a lower risk of cancer post cessation of alcohol compared to current drinkers if both groups being compared are representative of the population, yet what is observed is that just quitters have a higher risk of liver cancer than current drinkers. Prior studies have found that "former drinker" categories are often over-represented by former heavy drinkers and alcoholics[10]. If this is the case, then the risk of liver cancer for the cessation group *before *cessation would be higher than for the general group of current drinkers, and the increase in risk after cessation is an artefact of poorly matched groups. This is something that could have been controlled for but none of the studies presented any information on this. There are also reasons to doubt that for some of the studies the choice of control group is a reasonable representation of the population. Some of the studies removed certain individuals from the control groups who had past history of liver disease or certain illnesses making the risk of liver cancer in the control groups artificially low (see appendix 1 for details). There is not enough information about the control and treatment groups to test whether there are any important systematic differences between them that may explain why an increase in risk is observed for just quitters compared to current drinkers but serious doubts remain about how well they have been matched. There is also not enough information to assess how well either the control or treatment groups represent the wider population to be able to make an assessment of the external validity of the results. This implies that the results of the included studies and thus also this meta-analysis have a limited interpretation to that of the studied samples. The wider implications for the general population are debatable.

In order to account for this uncertainty and summarise the current position of the literature, a preliminary meta-analysis has been performed on two different forms of the same data to assess if there was any correspondence between the studies on the shape of the dose-response relationship of alcohol cessation and liver cancer. The first uses current drinkers as the reference category and calculates the dose-response relationship assuming current drinkers are the relevant reference group. The second deals with the potential overrepresentation of former heavy drinkers in the cessation group and uses just quit drinking as the reference category. Although this has the downside of reducing the sample size, it was expected to allow for a more accurate calculation of how risk declines after cessation of alcohol consumption.

The results where current drinkers were used as the reference group found only that recent quitters have much higher risk of liver cancer than current drinkers. Using former drinkers who had just quit as the reference group found a statistically significant downward trend in risk of liver cancer over time post cessation. This is the preferred estimate as it appears that it is based on a much better match between treatment and control groups. To answer the question at hand it is paramount that both treatment and control groups are representative of each other. If they differ in a significant way that biases the results it is impossible to draw robust conclusions about how risk changes after cessation of alcohol consumption. It seems that former drinkers in these studies have worse health than current drinkers and given that all of the former drinkers in these studies were at one time "just quitters" it then appears more sensible to use just quitters as the preferred control group. The rate of risk decline using just quitters as the reference group is quite robust to specification and common across the studies.

**Figure 4:**
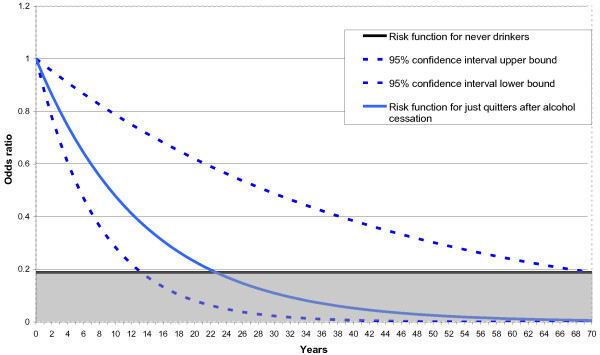
**Illustration: Risk decline of liver cancer post cessation of alcohol consumption compared to just quitters**.

By way of illustration, the findings from the meta-analysis tentatively suggest that for each year of cessation the risk of liver cancer declines by about 6-7% i.e. there is exponential decay. In order to calculate the length of time until increased risk of liver cancer has fallen to that of never drinkers, the relative risk of former drinkers compared to never drinkers is required. To ensure internal consistency of the estimates (acknowledging that a more precise and externally valid estimate is probably available in the literature), supplementary meta-analysis, not shown here, finds that the average odds ratio between just quit drinking and never drinkers is 0.19, significantly different from 1. Using the results from model 6 to illustrate what the current evidence suggests, it is estimated that it would take about 23 years (with correspondingly large 95% confidence interval of 14-70 years i.e. a confidence interval spanning 56 years) for the risk of liver cancer of quitters of alcohol to fall to that of never drinkers. This illustrative example based on the meta-analysis is shown diagrammatically in Figure 4.

## Conclusions

The rate of risk decline estimated here has great statistical uncertainty. It is also difficult to interpret, as the interpretation depends on whether the trend is the same for all groups or varies by key factors e.g. smoking, past alcohol consumption patterns, gender and age. The current extent of the literature means it is not known if this trend does vary by these key factors. It appears that the trend estimate found here is for a subgroup of the population but it is not possible to say what this sub-population looks like. There is indicative evidence that the treatment groups appear to be dissimilar to current drinkers and exhibit the patterns observed in heavy drinkers. Due to these issues, the results presented here should be interpreted with caution.

Many questions remain and more research is needed in this important area. Future research looking into the cessation effect of alcohol consumption on liver cancer will need to consider more seriously how well matched their treatment and control groups are, especially with regard to behaviours and factors associated with liver cancer outcomes. Care also needs to be taken in allowing interpretation of the external validity of the findings, either by controlling for characteristics so that broader conclusions can be drawn or by using sampling in a way that more accurately represents the wider population. The question at hand here also requires a long follow up as it is indicated in this study that the rate of decay in risk occurs over a substantial time period.

Given the difficult nature of studying alcohol intake's effect on human outcomes and the difficulty of separating the choice from the chooser, an effective study may need to make use of a policy experiment rather than just observe people's behaviour. The policy would have to be aimed at reducing intake of alcohol. An experiment large enough with a well matched and representative control group, ideally chosen at random would present both the ability to effectively assess the effectiveness of the policy and the effect of reduced alcohol intake on various outcomes (if the policy was effective of course).

## Competing interests

The authors report no conflicts of interest with people or organisations that could inappropriately influence the work. The authors did not receive any outside assistance writing this manuscript.

## Authors' contributions

BA carried out the initial systematic literature review and drafted this particular section of the manuscript. GH independently verified the findings of the literature review, performed the statistical analysis and drafted the manuscript. JJ and UG participated in the conception and design of the study and participated in the write-up of the manuscript. All authors read and approved the final manuscript.

## Pre-publication history

The pre-publication history for this paper can be accessed here:

http://www.biomedcentral.com/1471-2407/11/446/prepub

## Supplementary Material

Additional file 1 **Detailed findings from the systematic review**. A descriptive overview of the current literature examining the effect of alcohol cessation on liver cancer, including the raw data used in the meta-analysis. Click here for file

## References

[B1] Yu MC Mack T Hanisch R Peters RL Henderson BE Pike MC Hepatitis, alcohol-consumption, cigarette-smoking, and hepatocellular-carcinoma in los-angeles Cancer Research 1983 43 12 6077 6079 6315225

[B2] Austin H Delzell E Grufferman S Levine R Morrison AS Stolley PD Cole P A case-control study of hepatocellular-carcinoma and the hepatitis b-virus, cigarette-smoking, and alcohol-consumption Cancer Research 1986 46 2 962 966 3000590

[B3] Wang LY You SL Lu SN Ho HC Wu MH Sun CA Yang HI Chen CJ Risk of hepatocellular carcinoma and habits of alcohol drinking, betel quid chewing and cigarette smoking: a cohort of 2416 HBsAg-seropositive and 9421 HBsAg- seronegative male residents in Taiwan Cancer Causes & Control 2003 14 3 241 250 10.1023/A:1023636619477 12814203

[B4] Gutjahr E Gmel G Rehm J Relation between average alcohol consumption and disease: An overview European Addiction Research 2001 7 3 117 127 10.1159/000050729 11509842

[B5] Jarl J Gerdtham U-G Ludbrook A Petrie D On Measurement of Avoidable and Unavoidable Cost of Alcohol: An Application of Method for Estimating Costs Due To Prior Consumption International Journal of Environmental Research and Public Health 2010 7 7 2881 2895 10.3390/ijerph7072881 20717547 PMC2922734

[B6] Rehm J Patra J Popova S Alcohol drinking cessation and its effect on esophageal and head and neck cancers: A pooled analysis International Journal of Cancer 2007 121 5 1132 1137 10.1002/ijc.22798 17487833

[B7] Ogimoto I Shibata A Kurozawa Y Nose T Yoshimura T Suzuki H Iwai N Sakata R Fujita Y Ichikawa S Risk of death due to hepatocellular carcinoma among drinkers and ex-drinkers. Univariate analysis of JACC study data Kurume Med J 2004 51 1 59 70 10.2739/kurumemedj.51.59 15150901

[B8] Ozasa K Alcohol use and mortality in the Japan Collaborative Cohort Study for Evaluation of Cancer (JACC) Asian Pac J Cancer Prev 2007 8 Suppl 81 88 18260706

[B9] Hamling J Lee P Weitkunat R Ambuehl M Facilitating meta-analyses by deriving relative effect and precision estimates for alternative comparisons from a set of estimates presented by exposure level or disease category Statistics in Medicine 2008 27 7 954 970 10.1002/sim.3013 17676579

[B10] Johansson E Alho H Kiiskinen U Poikolainen K Abstaining from alcohol and labour market underperformance - Have we forgotten the 'dry' alcoholics? Alcohol and Alcoholism 2006 41 5 574 579 10.1093/alcalc/agl051 16855004

[B11] Egger M Smith GD Schneider M Minder C Bias in meta-analysis detected by a simple, graphical test British Medical Journal 1997 315 7109 629 634 10.1136/bmj.315.7109.629 9310563 PMC2127453

[B12] Greenland S Longnecker MP methods for trend estimation from summarized dose-response data, with applications to metaanalysis American Journal of Epidemiology 1992 135 11 1301 1309 1626547 10.1093/oxfordjournals.aje.a116237

[B13] Orsini n Bellocco r Greenland S Generalized least squares for trend estimation of summarized dose-response data The Stata Journal 2006 6 40 57

[B14] Goodman MT Moriwaki H Vaeth M Akiba S Hayabuchi H Mabuchi K Prospective cohort study of risk factors for primary liver cancer in Hiroshima and Nagasaki, Japan Epidemiology 1995 6 1 36 41 10.1097/00001648-199501000-00008 7888442

