# Supplementary material for: How the risk of liver cancer changes after alcohol cessation: A review and meta-analysis of the current literature
Source: BMC Cancer. 2011 Oct 13;11:446. doi: 10.1186/1471-2407-11-446 (PMC3229519; doi:10.1186/1471-2407-11-446)
Supplement: Additional file 1 — Detailed findings from the systematic review. A descriptive overview of the current literature examining the effect of alcohol cessation on liver cancer, including the raw data used in the meta-analysis. [file 1471-2407-11-446-S1.DOC]

**Additional file 1**

**Systematic review findings**

Six of the eleven studies included in this review are prospective cohort studies and five are case-control studies (**Table S1**). Two of the studies were conducted in Italy and the remaining nine in Japan. The study years were generally between 1965 and 2004 and were published between 1985 and 2007. Seven studies measured morbidity and three measured mortality from liver cancer. One study measured both morbidity and mortality from liver cancer[A4]. Five studies provided gender specific risk estimates whilst four did not. Two of the studies provided risk estimates for men only. The age of participants enrolled in the studies were generally between 40 and 79 years with the exception of three studies[A5-A7] which extended beyond this age group. Two of the prospective cohort studies[A1, A2] used data from the Japan Collaborative Cohort Study for the Evaluation of Cancer (JACC) and partially used the same sample. All the studies measured incidence cases.

Seven studies confirmed cases either totally or partially by histology. Four studies[A1, A2, A3, A5] did not provide information on how cases were diagnosed or confirmed. All case-control studies used hospital-based controls. Controls were out-patients or in-patients selected from the same hospitals as cases. All of the case-control studies used standard quality practice: data were collected in a similar manner for cases and controls; all cases were interviewed within six months of diagnosis (except in Tanaka *et al*.[A8] and Fukuda *et al*.[A9]); same interview schedule used for cases and controls; cases and controls interviewed during the same time period; same exclusion criteria for cases and controls. Generally, patients with history of liver disease were excluded from the control group. In Franceschi *et al*.[A6] those with diseases related to alcohol and tobacco use (such as respiratory diseases, peptic ulcer, lung cancer, head and neck cancer among others) or hepatitis viruses were purposively excluded from the control group. The study also excluded those with chronic diseases that could result in substantial lifestyle modification such as diabetes, cardiovascular and cerebrovascular diseases among others. This probably could have led to the recruitment of more healthy controls. With a control group that is not representative of the population at large the interpretation of the findings is uncertain as the external validity is unclear.

The study by Sakamoto *et al*.[A10] used two groups of controls. One was general hospital controls recruited from among first time visitors at an out-patient clinic whilst the other control group consisted of chronic liver disease patients without hepatocellular carcinoma recruited from both out-patient and in-patient clinics of two hospitals. However, patients with special types of chronic liver diseases were excluded from the controls. These were mainly primary and secondary biliary cirrhosis, autoimmune hepatitis and liver diseases resulting from parasitology, congestive heart failure or metabolic disorders.[A10]

With the case-control studies, sample size varied from 204-464 cases and 275-485 controls. One exception is the Donato *et al*.[A11] study which had a larger number of controls (824 controls). Cohort studies generally had a wide variation in sample size from 1,845-110,668 and registered 122-401 incidence cases in follow-up. Ozasa[A2] only reported person-years of follow-up (645,941 person-years). Tsukuma *et al*.[A4] had a smaller sample size and number of cases (917 cohort members, 54 liver cancer cases). Although Kono *et al*.[A5] had quite a large sample size (5,139 cohort members), the study registered fewer numbers of liver cancer cases (25 liver cancer mortalities) after more than 12 years of follow up. This could be because of the outcome used (mortality). All studies controlled for different potential confounders most commonly age and sex (**Table S2**).

In all the case-control studies, the definition of “former drinkers” was restricted to those who had quit drinking at least one year prior to the study or prior to diagnosis of liver cancer. On the contrary, five of the six cohort studies defined “former drinkers” as those who had quit drinking by the time of recruitment for the study. One cohort study contained no information on how “former drinkers” were defined with respect to time since cessation[A5]. This is an important variable to control for as it is desired that drinking patterns are not influenced by knowledge about the state of the liver. Not restricting former drinkers to those who have quit for at least one year prior to the study may mean that some participants’ behaviour may be influenced by information about their liver and therefore biasing the results.

Different statistical methods were used to compute the risk estimates. Four studies[A6, A8, A10, A11] used unconditional logistic regression to obtain the risk estimates (odds ratios). The Cox proportional regression model was used to compute the risk estimates in three studies (Tsukuma *et al*.[A4], *rate ratio*, Kato *et al*.[A7]; *relative risk*, Ogimoto *et al*.[A1]; *hazard ratio*). Ozasa[A2] also estimated the hazard ratio but the statistical method was not reported. In Kono *et al*.[A5], the Mantel-Haenszel procedure for incidence density data was used to statistically assess the adjusted death rates for the different drinking categories. Goodman *et al*.[A3] used Poisson regression for analysis and estimation of relative risk.

**Table S3** summarises the extracted data used for the meta-analysis with recalculated confidence intervals. All papers show a higher risk of liver cancer for just quitters compared to current drinkers. **Table S2** highlights how there is a mixed lack of controls for diabetes, smoking and importantly past drinking behaviour which could possibly explain this phenomenon.

**Table S1 – Study characteristics in studies of reduction in risk of liver cancer following drinking cessation**

| **Study** | **Country and study years** | **Type of study** | **Study area** | **Mortality/ morbidity** | **Gender specific calculations** | **Age** | **Gender cases (% male)** | **Sample characteristics regarding alcohol consumption** |
| --- | --- | --- | --- | --- | --- | --- | --- | --- |
| **Kono *et al*. 1985** | Japan,  1965-1977 | Prospective cohort | Liver | Mortality | Yes (men only) | 25-70 | 100 | Incidence cases |
| **Kato *et al*. 1992** | Japan,  1987-1990 | Prospective cohort | Liver | Morbidity | No | ≥16 | N/A | Incidence cases |
| **Tanaka *et al*. 1992** | Japan,  1985-1989 | Case-control | Liver | Morbidity | Yes | 40-69 | 82 | Incidence cases |
| **Fukuda *et al*. 1993** | Japan,  1986-1992 | Case-control | Liver | Morbidity | Yes | 40-69 | 78 | Incidence cases |
| **Tsukuma *et al*. 1993** | Japan,  1987-1991 | Prospective Cohort | Liver | Morbidity/  Mortality | No | 40-69 | N/A | Incidence cases |
| **Goodman *et al*. 1995** | Japan,  1978-1989 | Prospective cohort | Liver | Morbidity | Yes (men only) | N/A | N/A | Incidence cases |
| **Donato *et al*. 2002** | Italy,  1995-2000 | Case-control | Liver | Morbidity | Yes | 40-75 | 81.9 | Incidence cases |
| **Ogimoto *et al*. 2004** | Japan,  1988-1999 | Prospective cohort | Liver | Mortality | Yes | 40-79 | N/A | Japan Collaborative Cohort Study for Evaluation of Cancer |
| **Franceschi *et al*. 2006** | Italy,  1999-2002 | Case-control | Liver | Morbidity | No | 40-84 | 79.9 | Incidence cases |
| **Sakamoto *et al*. 2006** | Japan,  2001-2004 | Case-control | Liver | Morbidity | No | 40-79 | 67.5 | Incidence cases |
| **Ozasa 2007** | Japan | Prospective cohort | Liver | Mortality | Yes | N/A | N/A | Japan Collaborative Cohort Study for Evaluation of Cancer |

Notes: N/A = not available

**Table S2 – Quality characteristics of studies of reduction in risk of liver cancer following drinking cessation**

| **Study** | **Histological confirmation of cases** | **Source of controls** | **Sample size (case/control)** | **Response rate case/control (%)** | **Matched case-control study (matching factors)** | **Controlling for potential confounders** | **Statistical analysis** | **Period until classified as a former drinker** |
| --- | --- | --- | --- | --- | --- | --- | --- | --- |
| **Kono *et al*. 1985** | N/A | N/A | 5139 male Japanese physicians in cohort  25 cases of liver cancer | 49 | N/A | Yes (A, S) | Adjusted death rates using Mantel Haenszel procedure for incidence data | N/A |
| **Kato *et al*. 1992** | Yes | N/A | 1,845 cohort members  with de-compensated  liver cirrhosis, 122 liver cancer cases | N/A | N/A | Yes (A, G) | Cox proportional hazards regression | 0 years |
| **Tanaka *et al*. 1992** | 40% histological, 57.8% angiographic findings | Hospital | 204/410 | N/A | No | Yes (A, G) | Unconditional logistic regression | 1 year |
| **Fukuda *et al*. 1993** | (75.3% for males, 81.5% for females | Hospital | 368/485 | 99/100 | Yes (A, G, R, DoA) | N/A | Conditional logistic regression | 1 year |
| **Tsukuma *et al*., 1993** | 53.7% of cases | N/A | 917 persons in cohort,  54 liver cancer cases | N/A | N/A | Yes (A, G, S, SoD, SAF, HV) | Cox proportional-hazards regression | 0 years |
| **Goodman *et al*. 1995** | N/A | N/A | 36,133 persons in cohort, 242 liver cancer cases | 72.5 | N/A | Yes (A, G, R, Atb, RDL) | Poisson regression | 0 years |
| **Donato *et al*. 2002** | 84.7% of cases | Hospital | 464/824 | 93.5/96.1 | Yes (A, G, H, DoA) | Yes (A, G, R, HBV, HCV) | Unconditional Logistic regression | 1 year |
| **Ogimoto *et al*. 2004** | N/A | N/A | 110,668 cohort members, 401 liver cancer deaths | N/A | N/A | Yes (A, G, LD) | Cox proportional-hazards model | 0 years |
| **Franceschi *et al*. 2006** | 78.2% of cases | Hospital | 229/431 | 87.7/93.3 | Yes (A, G) | Yes (A, G, SC, E, HBV, HCV) | Unconditional multiple logistic regression | 1 year |
| **Sakamoto *et al*. 2006** | (28.2% biopsy, 58.8% angiography | Hospital | GH controls, 209/275 CLD patients, 209/381 | GH controls (92/73) CLD patients (92/96) | No | Yes (A, G, S, HBV, HCV) | Unconditional logistic regression | 1 year |
| **Ozasa 2007** | N/A | N/A | Only person years reported; 645,941 person years | N/A | N/A | Yes (A, SC) | Hazard ratio | 0 years |

Abbreviations: A, age; G, gender; E, education; S, smoking; R, residence; H, hospital of admission; GH, General Hospital ; CLD, chronic liver disease; SC, study centre; DoA, date of admission; HBV, hepatitis B virus; HCV, hepatitis C virus; SoD, stage of disease; HV, hepatitis virus markers; SAF, serum alpha-fetoprotein levels; Atb, age at time of bombing; RDL, radiation dose to the liver; LD, History of liver disease; N/A, Not available

Table S3 - Dose and rescaled risk estimates extracted from systematic review with adjusted confidence intervals

|  |  | **Current drinkers as reference group** | | | |  | **Recent quitters as reference group** | | | |
| --- | --- | --- | --- | --- | --- | --- | --- | --- | --- | --- |
|  |  |  |  | Confidence interval (95%) | |  |  |  | Confidence interval (95%) | |
| **Study** | Gender | Dose | Odds ratio | Low. bound | Up. bound |  | Dose | Odds Ratio | Low. bound | Up. bound |
| Donato et al. 2002 | Male | 0 | 1 |  |  |  | 0 | 1.0 |  |  |
|  |  | 3 | 5 | 2.9 | 8.6 |  | 5 | 0.8 | 0.4 | 1.7 |
|  |  | 8 | 4 | 2.2 | 7.3 |  | 10 | 0.3 | 0.1 | 1.0 |
|  |  | 13 | 1.6 | 0.6 | 4.4 |  | 15 | 0.3 | 0.1 | 0.7 |
|  |  | 18 | 1.4 | 0.6 | 3.2 |  |  |  |  |  |
|  | Female | 0 | 1 |  |  |  | 0 | 1.0 |  |  |
|  |  | 3 | 3 | 0.6 | 15.1 |  | 5 | 0.9 | 0.1 | 7.9 |
|  |  | 8 | 2.7 | 0.5 | 14.1 |  | 10 | 0.6 | 0.0 | 9.2 |
|  |  | 13 | 1.9 | 0.2 | 18.6 |  | 15 | 2.9 | 0.3 | 30.0 |
|  |  | 18 | 8.6 | 1.3 | 56.4 |  |  |  |  |  |
| Osaza et al. 2007 | Male | 0 | 1 |  |  |  | 0 | 1.0 |  |  |
|  |  | 2.5 | 4.3 | 2.6 | 7.0 |  | 8 | 1.2 | 0.6 | 2.3 |
|  |  | 10 | 5.1 | 3.3 | 7.9 |  | 18 | 0.6 | 0.3 | 1.4 |
|  |  | 20 | 2.7 | 1.4 | 5.2 |  |  |  |  |  |
|  | Female | 0 | 1 |  |  |  | 0 | 1.0 |  |  |
|  |  | 2.5 | 1.9 | 0.3 | 14.0 |  | 8 | 4.8 | 0.5 | 41.3 |
|  |  | 10 | 9.1 | 3.5 | 23.5 |  | 18 | 1.2 | 0.1 | 19.8 |
|  |  | 20 | 2.3 | 0.3 | 17.2 |  |  |  |  |  |
| Franceschi et al. 2006 | Both | 0 | 1 |  |  |  | 0 | 1.0 |  |  |
|  |  | 3 | 7.5 | 2.7 | 21.2 |  | 10 | 0.4 | 0.1 | 1.3 |
|  |  | 13 | 3 | 1.4 | 6.6 |  |  |  |  |  |
| Goodman et al. 1995 | Male | 0 | 1 |  |  |  | 0 | 1.0 |  |  |
|  |  | 5 | 8 | 4.3 | 14.9 |  | 9 | 0.3 | 0.1 | 0.7 |
|  |  | 13.5 | 2.1 | 1.0 | 4.4 |  | 14 | 0.1 | 0.0 | 0.4 |
|  |  | 18.5 | 1 | 0.4 | 2.7 |  |  |  |  |  |

References cited in additional file 1:

A1. Ogimoto I, Shibata A, Kurozawa Y, Nose T, Yoshimura T, Suzuki H, Iwai N, Sakata R, Fujita Y, Ichikawa S *et al*: **Risk of death due to hepatocellular carcinoma among drinkers and ex-drinkers. Univariate analysis of JACC study data**. *Kurume Med J* 2004, **51**(1):59-70.

A2. Ozasa K: **Alcohol use and mortality in the Japan Collaborative Cohort Study for Evaluation of Cancer (JACC)**. *Asian Pac J Cancer Prev* 2007, **8 Suppl**:81-88.

A3. Goodman MT, Moriwaki H, Vaeth M, Akiba S, Hayabuchi H, Mabuchi K: **Prospective cohort study of risk factors for primary liver cancer in Hiroshima and Nagasaki, Japan**. *Epidemiology* 1995, **6**(1):36-41.

A4. Tsukuma H, Hiyama T, Tanaka S, Nakao M, Yabuuchi T, Kitamura T, Nakanishi K, Fujimoto I, Inoue A, Yamazaki H *et al*: **Risk factors for hepatocellular carcinoma among patients with chronic liver disease**. *N Engl J Med* 1993, **328**(25):1797-1801.

A5. Kono S, Ikeda M, Tokudome S, Yoshimura T, Nishizumi M, Kuratsune M: **Alcohol and cancer in male Japanese physicians**. *J Cancer Res Clin Oncol* 1985, **109**(1):82-85.

A6. Franceschi S, Montella M, Polesel J, La Vecchia C, Crispo A, Dal Maso L, Casarin P, Izz F, TommaSi LG, Chemin I *et al*: **Hepatitis viruses, alcohol, and tobacco in the etiology of hepatocellular carcinoma in Italy**. *Cancer Epidemiology Biomarkers & Prevention* 2006, **15**(4):683-689.

A7. Kato I, Tominaga S, Ikari A: **The risk and predictive factors for developing liver cancer among patients with decompensated liver cirrhosis**. *Jpn J Clin Oncol* 1992, **22**(4):278-285.

A8. Tanaka K, Hirohata T, Takeshita S, Hirohata I, Koga S, Sugimachi K, Kanematsu T, Ohryohji F, Ishibashi H: **Hepatitis B virus, cigarette smoking and alcohol consumption in the development of hepatocellular carcinoma: a case-control study in Fukuoka, Japan**. *Int J Cancer* 1992, **51**(4):509-514.

A9. Fukuda K, Shibata A, Hirohata I, Tanikawa K, Yamaguchi G, Ishii M: **A hospital-based case-control study on hepatocellular carcinoma in Fukuoka and Saga Prefectures, northern Kyushu, Japan**. *Jpn J Cancer Res* 1993, **84**(7):708-714.

A10. Sakamoto T, Hara M, Higaki Y, Ichiba M, Horita M, Mizuta T, Eguchi Y, Yasutake T, Ozaki I, Yamamoto K *et al*: **Influence of alcohol consumption and gene polymorphisms of ADH2 and ALDH2 on hepatocellular carcinoma in a Japanese population**. *International Journal of Cancer* 2006, **118**(6):1501-1507.

A11. Donato F, Tagger A, Gelatti U, Parrinello G, Boffetta P, Albertini A, Decarli A, Trevisi P, Ribero ML, Martelli C *et al*: **Alcohol and hepatocellular carcinoma: the effect of lifetime intake and hepatitis virus infections in men and women**. *Am J Epidemiol* 2002, **155**(4):323-331.
